# Association of Low Family Income With Lung Function Among Children and Adolescents: Results of the J-SHINE Study

**DOI:** 10.2188/jea.JE20170220

**Published:** 2019-02-05

**Authors:** Airi Amemiya, Takeo Fujiwara

**Affiliations:** 1 Department of Social Medicine, National Research Institute for Child Health and Development, Tokyo, Japan; 2 Department of Global Health Promotion, Tokyo Medical and Dental University (TMDU), Tokyo, Japan

**Keywords:** low family income, lung function, children, adolescents, forced expiratory volume, Japan, poverty

## Abstract

**Background:**

The respiratory tract of children in low-income families is more likely to be exposed to toxins, which may lead to poor lung function. The purpose of this study was to elucidate the impact of low household income on lung function among children and adolescents in Japan.

**Methods:**

We analyzed a population-based sample of 1,224 children aged 5 to 17 years old from the Japanese Study on Stratification, Health, Income, and Neighborhood (J-SHINE). Forced expiratory volume in 1 s (FEV1) and in 6 s (FEV6) was measured. Annual household income and other potential covariates were assessed through a questionnaire. Low household income was defined as less than 3 million yen (approximately 27,000 United States dollars [USD]) per year. Multivariate regression analysis was used to adjust for potential covariates.

**Results:**

We observed statistically significantly lower FEV1:FEV6 ratio with children in lowest-income families compared with those in highest-income families, after adjusting for child’s age and sex (coefficient = −0.082; 95% confidence interval [CI], −0.131 to −0.034). After adjusting for other covariates, including parental smoking status and parental diagnosis of asthma, a similar trend remained (coefficient = −0.054; 95% CI, −0.109 to 0.001).

**Conclusions:**

Children in low-income families showed significantly lower lung function than those in high-income families. Prevention and early intervention are necessary to help the development of lung function among children living in low-income families.

## INTRODUCTION

Childhood asthma remains one of the most common chronic diseases worldwide. The International Study of Asthma and Allergies in Childhood found that approximately 14% of children worldwide were likely to have had asthmatic symptoms in 2009.^1^ The prevalence appears to be increasing in both developing and developed countries^1^; for example, 10.5% to 18.2% of children were affected in Japan in 2011.^2^ Socioeconomic disadvantage is an important determinant of childhood asthma^3^^,^^4^ and has been reported to be associated with asthma among children in developed countries.^4^ Other longitudinal studies showed that socioeconomic disadvantage was also associated with later asthma and low lung function in children.^5^^,^^6^

Most of the studies that investigated the association between low income and childhood asthma used parent-reported physician diagnosis or symptoms of asthma.^3^^,^^4^ For example, an Australian study showed children in low-income families had a two-fold increase in risk of asthma at age 14, especially in girls.^7^ The study assessed childhood asthma using a self-reported physician diagnosis or symptoms of asthma. Parent-reported physician diagnosis and symptoms are, however, subject to recall bias.^8^ Study participants might not accurately remember their diagnosis, or they might intentionally conceal their diagnosis or symptoms of asthma. Therefore, it is important to use objective measures of childhood lung function, such as forced expiratory volume (FEV), to assess the association with low income.

Several studies examined the association between socioeconomic status and childhood lung function in the United States,^9^^–^^11^ Canada,^12^ the United Kingdom,^6^ India,^13^ and Jamaica.^14^^,^^15^ Previous studies in the United States showed that low income was significantly associated with low lung function in males aged 8–17 years but not in females of the same age group.^9^^,^^16^ Other studies in the United States, Canada, and the United Kingdom showed that low socioeconomic status was associated with low lung function among children, assessing socioeconomic status using parental education and occupation.^6^^,^^10^^,^^12^

In 2014, 16.3% of children in Japan were living in relative poverty,^17^ which was higher than the average percentages of the 34 Organization for Economic Co-operation and Development (OECD) members in 2012.^18^ However, few studies have investigated the association between low income and childhood asthma or lung function in Japan. In this large population-based study, we examined the association between low household income and childhood lung function in Japan. Lung function was objectively measured using a spirometer.

## MATERIAL AND METHODS

### Study design

This study was conducted with a cross-sectional design using data from the Japanese Study on Stratification, Health, Income, and Neighborhood (J-SHINE). J-SHINE is an ongoing panel study of households of adults and children. It was designed to investigate the complex associations between social factors and health. The design and methods used in J-SHINE have been described elsewhere.^19^

**Figure 1.**
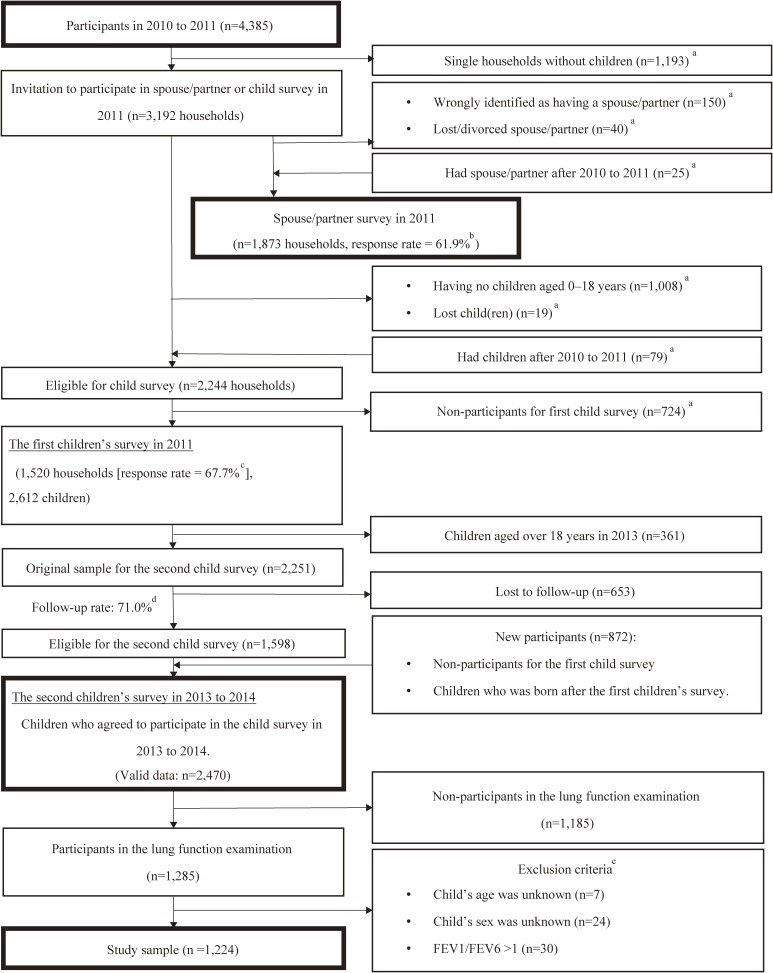
Sample flowchart. ^a^The unit was households. ^b^1,873 divided by 3,027. ^c^1,520 divided by 2,244. ^d^1,598 divided by 2,251. ^e^There was no overlap of the two missing variables for child’s age and sex, and FEV1/FEV6 >1.

In the wave 1 survey, conducted between July 2010 and February 2011, community-dwelling men and women aged 25 to 50 years living in four municipalities in and around Tokyo were probabilistically and randomly sampled from the Basic Resident Registration System. Of 8,408 adults eligible for the wave 1 survey, 4,385 participated (Figure 1). Among them, 3,192 adults with a spouse/partner or child were invited to participate in a spouse/partner or children’s survey between August and December 2011. Of 2,244 eligible households with both parents and children under the age of 18, 1,520 participated in the spouse/partner and children’s survey (the number of children was 2,612). In the second children’s survey, conducted between November 2013 and February 2014, participants who had reached the age of 18 were excluded (*n* = 361); among 2,251 children who participated in the wave 1 survey, 1,598 participated in the wave 2 survey (the follow-up rate was 71.0%). Those who did not participate in the survey and those who were born after the first survey were included in the second children’s survey (*n* = 872). As a result, the number of valid participants in the second children’s survey was 2,470. Data from these surveys were used in this study. The J-SHINE survey of children was conducted using a computer-assisted personal interview format, with responses given by their parents.

Of the 2,470 children, 1,285 (52.0%) participated in a lung function examination in 2013 (eTable 1). We excluded participants whose age (*n* = 7) or sex (*n* = 24) was unknown or whose forced expiratory volume in 1 s (FEV1) over FEV in 6 s (FEV1:FEV6) was >1 (*n* = 30); there was no overlap of the two missing variables for child’s age and sex and FEV1:FEV6 >1. A total of 1,224 eligible participants were analyzed. The study was approved by the ethics committee of the Graduate School of Medicine and Faculty of Medicine, The University of Tokyo. Consent for participation in the study and the agreement for publication was indicated by completing and returning the self-administered questionnaire.

### Measurements

Lung function was assessed as the ratio of FEV1:FEV6 using a calibrated dry spirometer (copd-6, Vitalograph^®^, Tokyo, Japan) according to the American Thoracic Society standards. A previous paper confirmed the good sensitivity and specificity of the handheld spirometer, and we believe that use of this handheld device would be adequate and most feasible in a population-based epidemiological study.^20^ Although the handheld spirometer measures FEV1 and FEV6, it is incapable of measuring forced vital capacity (FVC), which is required for calculating FEV1% (FEV1:FVC). Regardless of this limitation, FEV6 was used as an alternative measure for FVC as reports have shown that FEV6 obtained using this handheld spirometer can yield the actual value of measured FVC.^21^^–^^23^ The examination was considered acceptable when a beeping sound was produced, which indicated that an expiration of at least 6 s had been accomplished. Any attempt that was not associated with a beeping sound was regarded as unacceptable. If the first measurement could not be obtained, a second measurement was conducted. If the measurement value was obtained twice, the higher FEV1:FEV6 values were used in the analysis. After the caregiver agreed to have his/her child’s lung function examined, the child was instructed to perform the forced expiratory maneuvers.

Annual household income was assessed through a questionnaire that was filled out by the primary caregivers, and the amount was divided into five categories: less than 3 million Japanese yen (JPY) (approximately 27,000 United States’ dollars [USD]), 3 to less than 5 million JPY (27,000–45,000 USD), 5 to less than 7.5 million JPY (45,000–68,000 USD), 7.5 to less than 10 million JPY (68,000–90,000 USD), and 10 or more million JPY (more than 90,000 USD). Low annual household income was defined as less than 3 million JPY (equivalent to 27,000 USD) per year, based on 50% of the median national household income.^17^ The questionnaire also asked about other basic demographic variables of the children, including sex, age, and number of siblings. The child’s height and weight were measured during the interview, body mass index (BMI) was calculated by dividing the weight in kilograms by the height squared in meters, and the z-score of the BMI was estimated using the World Health Organization’s (WHO’s) growth reference data for the age group of 5 to 19 years.^24^ The questionnaire also enquired about parental covariates, including sex, age, BMI, education attainment, employment status, smoking status, physician diagnosis of asthma in the parents, residence, and marriage status. We categorized maternal BMI into thin (BMI <18.5), normal (BMI 18.5–24.9), overweight (BMI ≥25), and a dummy variable (missing values, *n* = 180). Parental educational attainment was divided into the three categories of high school or less, vocational or junior college, and college or more. Parental employment status was divided into the three categories of full-time job, part-time job, self-employed, and unemployed. Missing values were treated as dummy variables. The following analysis included participants with a single parent, and we treated their parental variables as a missing variable if there was no corresponding parent.

### Statistical analysis

The association between annual household income and children’s lung function was analyzed using multivariate regression analysis. In addition to an initial crude model adjusting for age and sex, three adjusted models were employed. Model 1 was further adjusted for demographic and social covariates, including children’s BMI,^25^ number of siblings,^26^ both parental education attainment^27^ and employment status,^28^ maternal BMI,^29^ and residence.^30^ Model 2 was further adjusted for parental smoking status, because parental smoking is a possible mediator between low income and poor lung function among children.^31^ Model 3 was further adjusted for physician diagnosis of asthma in the parents in addition to the covariates in model 2, in order to account for a family history of asthma.^32^ We stratified sex and age groups (5–12 and 13–17 years) in subgroup analysis because there could be sex differences in the development of asthma^33^ and the prevalence rate of asthma reduces in adolescence.^1^ Two-sided *P* < 0.05 was considered statistically significant. All of the analyses were conducted using Stata/MP v14.0 software (Stata Corp, College Station, TX, USA).

## RESULTS

Compared with non-participants, participants had a higher annual household income (*P* for trend = 0.002, which was calculated without subjects with missing variables in annual household income), greater number of siblings, older parents, lower level of educational attainment by the mother, lower proportion of mothers with a full-time job, and higher percentage of parents who are smokers (eTable 1).

Participant characteristics are shown in Table 1. The mean age of the children was 11.5 years, and 49.8% were male. The mean age of the fathers and mothers was 42.0 years and 40.5 years, respectively. The proportion of fathers who had a high school education or less was 20.4%, 23.9% did not have a full-time job, and 28.3% were current smokers. The proportion of mothers who had a high school education or less was 24.4%, 41.0% were unemployed, and 10.8% were current smokers. Almost all (95.3%) of the parents were married or had a partner. The proportion of fathers and mother diagnosed with asthma was 4.8% and 5.4%, respectively. The percentage of families earning less than 3 million JPY was 4.1%. Participant characteristics stratified by sex and age groups are shown in eTable 2.

**Table 1.** Sociodemographic and asthma risk factor characteristics (*n* = 1,224)

		Total		observations	mean	SD	min	max

		*n*	%					
Child								
Age				1,224	11.5	3.2	5	17
Sex	Male	610	49.8					
	Female	614	50.2					
BMI	Thin	45	3.7					
	−2SD∼+1SD	1031	84.2					
	Overweight	148	12.1					
Number of siblings	0	161	13.2					
	1	653	53.4					
	≥2	410	33.5					
Physician diagnosis of asthma		99	8.1					
Mother								
Age				1,083	40.5	5.1	16	52
Educational attainment	High school or less	299	24.4					
	Vocational/junior college	565	46.2					
	college or more	241	19.7					
	Missing	119	9.7					
Employment status	Full-time job	113	9.2					
	Part-time job	412	33.7					
	Self-employed	88	7.2					
	Unemployed	502	41.0					
	Missing	109	8.9					
BMI	<18.5	126	10.3					
	18.5–24.9	806	65.9					
	≥25	112	9.2					
	Missing	180	14.7					
Smoking status	Smoking	132	10.8					
	Quit	241	19.7					
	Never	711	58.1					
	Missing	140	11.4					
Physician diagnosis of asthma		66	5.4					
Father								
Age				961	42.0	5.6	26	61
Educational attainment	High school or less	250	20.4					
	Vocational/junior college	226	18.5					
	college or more	593	48.5					
	Missing	155	12.7					
Employment status	Full-time job	932	76.1					
	Part-time job	39	3.2					
	Self-employed	96	7.8					
	Unemployed	13	1.1					
	Missing	144	11.8					
Smoking status	Smoking	346	28.3					
	Quit	332	27.1					
	Never	295	24.1					
	Missing	251	20.5					
Physician diagnosis of asthma		59	4.8					
Household								
Marriage status	Married/Partner	4	0.3					
	None	1167	95.3					
	Missing	53	4.3					
Annual household income (million Japanese yen)	<3	50	4.1					
	3–<5	176	14.4					
	5–<7	310	25.3					
	7–<10	250	20.4					
	>10	165	13.5					
	Missing	273	22.3					

BMI, body mass index; SD, standard deviation.

Table 2 shows the coefficients of annual household income for FEV1:FEV6. The crude model showed statistically significantly lower FEV1:FEV6 for children in the lowest-income families (less than 3 million JPY) compared with those in the highest-income families (more than 10 million JPY) after adjusting for child’s age and sex (coefficient = −0.082; 95% confidence interval [CI], −0.131 to −0.034). After adjusting for other covariates, a similar trend remained (coefficient = −0.050; 95% CI, −0.104 to 0.004) in model 1. After adjusting for parental smoking status (model 2) and parental diagnosis of asthma (model 3), a similar trend remained (coefficient = −0.053; 95% CI, −0.108 to 0.002 and coefficient = −0.054; 95% CI, −0.109 to 0.001, respectively).

**Table 2.** The coefficients of income on FEV1/FEV6 by multiple regression analysis (*n* = 1,224)

Income (million JPY)	*N*	Crude		Model 1		Model 2		Model 3	
		Adjusted age and sex		Crude + covariates		Model 1 + parental smoking status		Model 2 + parental diagnosis of asthma	
			
		Coef.	95% CI	Coef.	95% CI	Coef.	95% CI	Coef.	95% CI
<3	50	**−0.082**	**−0.131, −0.034**	−0.050	−0.104, 0.004	−0.053	−0.108, 0.002	−0.054	−0.109, 0.001
3–<5	176	−0.009	−0.042, 0.024	0.006	−0.029, 0.042	0.003	−0.033, 0.040	0.004	−0.033, 0.040
5–<7	310	−0.004	−0.033, 0.025	0.015	−0.017, 0.046	0.013	−0.019, 0.044	0.014	−0.017, 0.046
7–<10	250	−0.006	−0.036, 0.024	0.004	−0.027, 0.036	0.002	−0.029, 0.034	0.004	−0.028, 0.036
>10	165	Ref.		Ref.		Ref.		Ref.	
missing	273	0.004	−0.026, 0.034	0.023	−0.012, 0.058	0.019	−0.016, 0.054	0.019	−0.016, 0.054

**Bold: *P* < 0.05.**

CI, confidence interval; Coef, coefficient; FEV, forced expiratory volume; JPY, Japanese yen; Ref, reference.

Covariates are children’s BMI, number of siblings, both parental education attainment and employment status, maternal BMI, and residence.

Among males, the crude model showed statistically significantly lower FEV1:FEV6 in children from the lowest-income families compared with in those from the highest-income families after adjusting for child’s age (coefficient = −0.110; 95% CI, −0.169 to −0.030; eTable 3). No significant association between income and FEV1:FEV6 was observed among females (eTable 4). As for age-stratified analysis, among the 5–12-year-old age group, the crude model showed statistically significantly lower FEV1:FEV6 in children from the lowest-income families compared with in those from the highest-income families after adjusting for child’s age and sex (coefficient = −0.094; 95% CI, −0.151 to −0.037; eTable 5). No significant association between income and FEV1:FEV6 was observed among 13–17-year-old children (eTable 6). The interaction terms of both income-sex (*P* = 0.011) and income-age (*P* = 0.047) were significant.

## DISCUSSION

We demonstrated that children in low-income families have significantly lower lung function in Japan. A similar trend remained after adjustment for sociodemographic covariates, parental smoking status, and parental physician diagnosis of asthma, suggesting the direct impact of living in a low-income household on the lung function of children. The stratified analysis by sex and age groups showed similar findings, especially in males and in the 5–12-year-old age group.

The result was consistent with recent studies that reported low socioeconomic status was associated with poor lung function in children in developed countries.^15^ Studies in the United States reported that low poverty index (defined as the ratio of family income to the federal poverty line) was associated with lower FEV1 and FVC in male children, and parental occupation and education was associated with lower FVC and FEV0.75 in children.^9^^,^^10^ A study in Canada reported that lower levels of parental occupation were associated with lower FEV1 and FVC in males only and were not associated with FEV1:FVC ratio in both sexes.^12^ This study adds to the literature by showing the association between low family income and FEV1:FEV6 in children in Japan. Notably, FEV1:FEV6 is an alternative measure for FEV1:FVC used in the diagnosis of airway obstruction.^34^

Several possible reasons could explain the association between living in a low-income household and lower lung function in young children. Children from low-income families tend to have more airway inflammation as a result of chronic exposure to toxins, such as house dust mite feces or traffic-related air pollution, although this study could not assess these possible mediators. House dust mite feces are more likely to accumulate in the houses of low-income families, which leads to the entry of fecal particles into the lungs.^35^ It is interesting to note that parental smoking could not account for the association between low income and poor lung function in children, although a previous study suggested that parental smoking status could explain the association.^31^ One possibility for this inconsistency is that we did not assess smoking quantity and whether smoking occurred indoors or outside the house. More precise evaluation of environmental tobacco smoke might show the detrimental impact on children’s lung function. In addition to a poor environment, less access to health services might also contribute to lower lung function among children in low-income families. Although the Japanese Universal Health Insurance System insures all residents,^36^ one of the major reasons cited for refraining from receiving medical services was economic hardship.^37^ Thus, low-income parents might be reluctant to seek medical attention for their children even if they recognize the child’s shortness of breath, coughing, or wheezing. A psychosocial pathway might be another possibility to explain the association between low-income and asthma. Chronic psychosocial distress due to financial strain^38^ may directly deteriorate childhood lung function through exaggerated airway inflammatory response.^39^^–^^43^ Acute psychosocial stress, such as witnessing of domestic violence or community crime, was also more common in children with low socioeconomic status and may lead to acute airway inflammatory responses.^39^^,^^44^^–^^46^

As for sex, we found that a low-income household was associated with lower lung function in males but not in females. The result was consistent with other studies, which reported that low socioeconomic status was associated with low FEV1 and FVC only in male children, although it was not consistent with a study that reported parental educational level was associated with lower FEV1 only among females in North America.^9^^,^^12^ The discrepancy may be due to differences in growing rate in lung function between females and males; that is, higher lung function may buffer the impact of low household income, resulting in different susceptibilities to socioeconomic circumstances.^47^ As for age groups, low-income household was associated with lower lung function in elementary school children (age 5–12) but not in junior high school children (age 13–17) in this study. The non-significance in junior high school children might be due to lung development or the small sample size. The 95% CIs of the lowest-income group with lower lung function were wide, possibly because the number of children in the lowest-income group was small. A larger sample would be necessary to identify sex and age groups that are vulnerable to low lung function for low household income.

This study has several limitations. First, the response rate was low. The low response rate may lead to underestimation of the prevalence of low lung function in children; a study reported that the prevalence rate of respiratory symptoms and diseases was higher in non-responders than in responders of the survey.^48^ Second, as this was a cross-sectional study, the association might be explained by reverse causation bias; for example, parents whose child’s lung function is low might have less time to earn money because they have to care for the child.

Despite these limitations, our findings highlight the need to monitor socioeconomic status in children. According to the WHO, monitoring and addressing socioeconomic status are important to reduce health inequalities.^49^ In Japan, the percentage of children living in relative poverty has been gradually increasing in the last three decades, which may have accounted for the increase in the prevalence of childhood asthma.^18^ Reducing the prevalence of relative poverty among children may contribute to preventing childhood asthma.

## APPENDIX A. SUPPLEMENTARY DATA

eTable 1. Characteristics sorted by non-participants and participants of lung function examination

eTable 2. Characteristics sorted by sex and age groups

eTable 3. The coefficients of income on FEV1/FEV6 by multiple regression analysis in males (n = 610)

eTable 4. The coefficients of income on FEV1/FEV6 by multiple regression analysis in females (n = 614)

eTable 5. The coefficients of income on FEV1/FEV6 by multiple regression analysis at age 5–12 years old (n = 741)

eTable 6. The coefficients of income on FEV1/FEV6 by multiple regression analysis at age 13–17 years old (n = 483)
