# Supplementary material for: Association of Low Family Income With Lung Function Among Children and Adolescents: Results of the J-SHINE Study
Source: J Epidemiol. 2019 Feb 5;29(2):50–6. doi: 10.2188/jea.JE20170220 (PMC6336726; doi:10.2188/jea.JE20170220)
Supplement: Supplementary file 1 [file je-29-050-s001.pdf]

Supplement

eTable 1. Characteristics sorted by non-participants and participants of lung function examination

			Non-participants (n=1,185)		Participants (n=1,285)		
			mean	SD	mean	SD	¶p
Child	Age[a]		11.9	3.3	11.6	3.2	0.201
Parents	Father's age[b]		37.2	0.2	42.0	0.2	<0.001
	Mother's age[c]		35.8	0.2	40.5	0.2	<0.001

			Non-participants		Participants		
			n	%	n	%	p*
Child							
	Sex	Male	613	51.7	628	48.9	0.172
		Female	553	46.7	633	49.3	
		Missing	19	1.6	24	1.9	
	BMI	Thin	5	0.4	45	3.5	<0.001
		-2SD~+1SD	1,153	97.3	1,089	84.7	
		Overweight	27	2.3	151	11.8	
	Number of siblings	0	239	20.2	187	14.6	<0.001
		1	638	53.8	674	52.5	
		2+	308	26.0	424	33.0	
	Physician diagnosis of asthma		90	7.6	102	7.9	0.189
Mother							
	Educational attainment	High school or less	230	19.4	314	24.4	<0.001
		Vocational/junior college	487	41.1	598	46.5	
		College or more	352	29.7	249	19.4	

		Missing	116	9.8	124	9.6	
	Employment status	Full-time job	189	15.9	119	9.3	<0.001
		Part-time job	187	15.8	439	34.2	
		Self-employed	44	3.7	91	7.1	
		Unemployed	645	54.4	522	40.6	
		Missing	120	10.1	114	8.9	
	BMI	<18.5	132	11.1	131	10.2	0.013
		≥18.5, <25	758	64.0	852	66.3	
		≥25	79	6.7	116	9.0	
		Missing	216	18.2	186	14.5	
	Smoking status	Smoking	70	5.9	141	11.0	<0.001
		Quit	250	21.1	253	19.7	
		Never	686	57.9	747	58.1	
		Missing	179	15.1	144	11.2	
	Physician diagnosis of asthma		76	6.4	74	5.8	0.005
Father							
	Educational attainment	High school or less	244	20.6	264	20.5	0.190
		Vocational/junior college	212	17.9	244	19.0	
		College or more	609	51.4	617	48.0	
		Missing	120	10.1	160	12.5	
	Employment status	Full-time job	906	76.5	981	76.3	0.193
		Part-time job	58	4.9	41	3.2	
		Self-employed	90	7.6	101	7.9	
		Unemployed	9	0.8	14	1.1	
		Missing	122	10.3	148	11.5	
	Smoking status	Smoking	277	23.4	357	27.8	0.012
		Quit	325	27.4	353	27.5	

		Never	352	29.7	315	24.5	
		Missing	231	19.5	260	20.2	
	Physician diagnosis of asthma		72	6.1	63	4.9	0.430
Household							
	Marriage status	Married/Partner	13	1.1	4	0.3	0.001
		None	1,146	96.7	1,226	95.4	
		Missing	26	2.2	55	4.3	
	Annual household income	<3	41	3.5	52	4.0	0.002
	(million Japanese yen)	3–<5	228	19.2	186	14.5	
		5–<7	329	27.8	327	25.4	
		7–<10	207	17.5	260	20.2	
		>10	117	9.9	170	13.2	
		Missing	263	22.2	290	22.6	

BMI, body mass index; SD, standard deviation.

[*] p from chi-square test for categorical variables and *p* from *t*-test for continuous variables

[a] Children's age was calculated using those without missing data for ages (non-participants, n=225; participants, n=1278).

[b] Father's age was calculated using those without missing data for ages (non-participants, n=949; participants, n=1010).

[c] Mother's age was calculated using those without missing data for ages (non-participants, n=1003; participants, n=1139).

eTable 2. Characteristics sorted by sex and age groups

		Sex				Age groups			
		Male (n=610)		Female (n=614)		5–12 years old (n 741)		13–17 years old (n=483)	
		mean	SD	mean	SD	mean	SD	mean	SD
Child	Age	11.5	3.2	11.6	3.2	9.3	1.8	14.9	1.4
Parents	Father's age	42.1	5.5	42.0	5.7	40.2	5.3	45.1	4.7
	Mother's age	40.6	5.1	40.5	5.2	38.5	4.7	43.5	4.2

			Sex				Age groups			
			Male (n=610)		Female (n=614)		5–12 years old (n 741)		13–17 years old (n=483)	
			n	%	n	%	n	%	n	%
Child										
	Sex	Male	610	100.0	0	0.0	368	49.7	242	50.1
		Female	0	0.0	614	100.0	373	50.3	241	29.9
	BMI	Thin	27	4.4	18	2.9	34	4.6	11	2.3
		-2SD~+1SD	479	78.5	552	89.9	602	81.2	429	88.8
		Overweight	104	17.1	44	7.2	105	14.2	43	8.9
	Number of siblings	0	88	14.4	73	11.9	100	13.5	61	12.6
		1	307	50.3	346	56.4	373	50.3	280	58.0
		2+	215	35.3	195	31.8	268	36.2	142	29.4
	Physician diagnosis of asthma		65	10.7	34	5.5	70	9.5	29	6.0
Mother										

	Educational attainment	High school or less	148	24.3	151	24.6	189	25.5	110	22.8
		Vocational/junior college	285	46.7	280	45.6	324	43.7	241	49.9
		College or more	122	20.0	119	19.4	152	20.5	89	18.4
		Missing	55	9.0	64	10.4	76	10.3	43	8.9
	Employment status	Full-time job	56	9.2	466	75.9	65	8.8	48	9.9
		Part-time job	206	33.8	23	3.8	190	25.6	222	46.0
		Self-employed	34	5.6	41	6.7	47	6.3	41	8.5
		Unemployed	262	43.0	9	1.5	370	49.9	132	27.3
		Missing	52	8.5	75	12.2	69	9.3	40	8.3
	BMI	<18.5	74	12.1	52	8.5	77	10.4	49	10.1
		≥18.5, <25	397	65.1	409	66.6	485	65.5	321	66.5
		≥25	54	8.9	58	9.5	64	8.6	48	9.9
		Missing	85	13.9	95	15.5	115	15.5	65	13.5
	Smoking status	Smoking	68	11.2	186	30.3	85	11.5	47	9.7
		Quit	113	18.5	163	26.6	154	20.8	87	18.0
		Never	361	39.2	147	23.9	410	55.3	301	62.3
		Missing	68	11.2	118	19.2	92	12.4	48	9.9
	Physician diagnosis of asthma		39	6.4	34	5.5	41	5.5	25	5.2
Father										
	Educational attainment	High school or less	126	20.7	124	20.2	154	20.8	96	19.9
		Vocational/junior college	104	17.1	122	19.9	151	20.4	75	15.5
		College or more	307	50.3	286	46.6	334	45.1	259	53.6
		Missing	73	12.0	82	13.4	102	13.8	53	11.0
	Employment status	Full-time job	466	76.4	466	75.9	556	75.0	376	77.9
		Part-time job	16	2.6	23	3.8	26	3.5	13	2.7
		Self-employed	55	9.0	41	6.7	62	8.4	34	7.0
		Unemployed	4	0.7	9	1.5	5	0.7	8	1.7

		Missing	69	11.3	75	12.2	92	12.4	52	10.8
	Smoking status	Smoking	160	26.2	186	30.3	232	31.3	114	23.6
		Quit	169	27.7	163	26.6	183	24.7	149	30.9
		Never	148	24.3	147	23.9	185	25.0	110	22.8
		Missing	133	21.8	118	19.2	141	19.0	110	22.8
	Physician diagnosis of asthma		30	4.9	29	4.7	37	4.5	22	4.6
Household										
	Annual household income	<3	25	4.1	25	4.1	34	4.6	16	3.3
	(million Japanese yen)	3–<5	86	14.1	90	14.7	131	17.7	45	9.3
		5–<7	154	25.3	156	25.4	204	27.5	106	22.0
		7–<10	137	22.5	113	18.4	131	17.7	119	24.6
		>10	79	13.0	86	14.0	72	9.7	93	19.3
		Missing	129	21.2	144	12.5	169	22.8	104	21.5

BMI, body mass index; SD, standard deviation.

eTable 3. The coefficients of income on FEV1/FEV6 by multiple regression analysis in males (n=610)

		Crude<br>Adjusted age		Model 1<br>Crude+ covariates		Model 2<br>Model 1+ parental smoking status		Model 3<br>Model 2+parental diagnosis of asthma	
Income (million JPY)	n	Coef.	95% CI	Coef.	95% CI	Coef.	95% CI	Coef.	95% CI
<3	25	**-0.100**	**-0.169, -0.030**	-0.050	-0.131, 0.032	-0.055	-0.138, 0.027	-0.055	-0.137, 0.028
3–<5	86	0.000	-0.048, 0.048	0.016	-0.036, 0.068	0.013	-0.040, -0.066	0.012	-0.041, 0.066
5–<7	154	0.000	-0.042, 0.043	0.018	-0.029, 0.064	0.013	-0.034, 0.061	0.015	-0.033, 0.062
7–<10	137	0.006	-0.037, 0.048	0.013	-0.032, 0.059	0.008	-0.038, 0.054	0.010	-0.036, 0.056
>10	79	Ref.		Ref.		Ref.		Ref.	
missing	129	0.016	-0.028, 0.060	0.054	0.002, 0.106	0.048	-0.005, 0.101	0.049	-0.004, 0.102

Bold: p<0.05

CI, confidence interval; Coef, coefficient; FEV, forced expiratory volume; JPY, Japanese yen; Ref, reference.

Covariates are children's BMI, number of siblings, both parental education attainment and employment status, maternal BMI, and residence.

eTable 4. The coefficients of income on FEV1/FEV6 by multiple regression analysis in females (n=614)

		Crude<br>Adjusted age		Model 1<br>Crude+ covariates		Model 2<br>Model 1+ parental smoking status		Model 3<br>Model 2+parental diagnosis of asthma	
Income (million JPY)	n	Coef.	95% CI	Coef.	95% CI	Coef.	95% CI	Coef.	95% CI
<3	25	-0.066	-0.134, 0.001	-0.032	-0.107, 0.042	-0.031	-0.107, 0.045	-0.033	-0.110, 0.043
3–<5	90	-0.019	-0.065, 0.026	-0.009	-0.059, 0.041	-0.010	-0.061, 0.041	-0.008	-0.060, 0.043
5–<7	156	-0.009	-0.049, 0.031	0.006	-0.038, 0.049	0.006	-0.038, 0.050	0.007	-0.037, 0.051
7–<10	113	-0.019	-0.061, 0.024	-0.010	-0.054, 0.035	-0.009	-0.054, 0.036	-0.007	-0.052, 0.037
>10	86	Ref.		Ref.		Ref.		Ref.	
missing	144	-0.009	-0.049, 0.032	-0.008	-0.056, 0.040	-0.008	-0.057, 0.040	-0.010	-0.058, 0.039

Bold: p < 0.05

CI, confidence interval; Coef, coefficient; FEV, forced expiratory volume; JPY, Japanese yen; Ref, reference.

Covariates are children's BMI, number of siblings, both parental education attainment and employment status, maternal BMI, and residence.

eTable 5. The coefficients of income on FEV1/FEV6 by multiple regression analysis at age 5-12 years old (n=741)

		Crude<br>Adjusted age and sex		Model 1<br>Crude+ covariates		Model 2<br>Model 1+ parental smoking status		Model 3<br>Model 2+parental diagnosis of asthma	
Income (million JPY)	n	Coef.	95% CI	Coef.	95% CI	Coef.	95% CI	Coef.	95% CI
<3	34	**-0.094**	**-0.151, -0.037**	-0.055	-0.119, 0.008	-0.063	-0.128, 0.002	-0.064	-0.128, 0.001
3–<5	131	-0.004	-0.045, 0.036	0.019	-0.024, 0.062	0.015	-0.029, 0.059	0.015	-0.029, 0.059
5–<7	204	-0.003	-0.041, 0.035	0.019	-0.021, 0.060	0.016	-0.025, 0.057	0.017	-0.025, 0.058
7–<10	131	-0.005	-0.045, 0.035	0.009	-0.034, 0.051	0.008	-0.035, 0.050	0.008	-0.034, 0.051
>10	72	Ref.		Ref.		Ref.		Ref.	
missing	169	0.016	-0.022, 0.055	0.035	-0.01, 0.079	0.030	-0.015, 0.075	0.029	-0.016, 0.074

Bold: *p*<0.05

CI, confidence interval; Coef, coefficient; FEV, forced expiratory volume; JPY, Japanese yen; Ref, reference.

Covariates are children's BMI, number of siblings, both parental education attainment and employment status, maternal BMI, and residence.

eTable 6. The coefficients of income on FEV1/FEV6 by multiple regression analysis at age 13-17 years old (n=483)

		Crude Adjusted age and sex		Model 1 Crude+ covariates		Model 2 Model 1+ parental smoking status		Model 3 Model 2+parental diagnosis of asthma	
Income (million JPY)	n	Coef.	95% CI	Coef.	95% CI	Coef.	95% CI	Coef.	95% CI
<3	16	-0.049	-0.139, 0.041	-0.016	-0.119, 0.088	-0.018	-0.122, 0.087	-0.017	-0.124, 0.090
3–<5	45	-0.016	-0.077, 0.044	-0.009	-0.078, 0.060	-0.009	-0.079, 0.061	-0.008	-0.079, 0.063
5–<7	106	0.003	-0.044, 0.050	0.015	-0.038, 0.068	0.014	-0.039, 0.068	0.019	-0.035, 0.072
7–<10	119	-0.006	-0.052, 0.040	-0.001	-0.050, 0.049	-0.003	-0.053, 0.047	-0.001	-0.051, 0.050
>10	93	Ref.		Ref.		Ref.		Ref.	
missing	104	-0.012	-0.059, 0.036	0.024	-0.036, 0.084	0.025	-0.036, 0.086	0.028	-0.033, 0.089

Bold: *p*<0.05

CI, confidence interval; Coef, coefficient; FEV, forced expiratory volume; JPY, Japanese yen; Ref, reference.

Covariates are children’s BMI, number of siblings, both parental education attainment and employment status, maternal BMI, and residence.
